# How older adults self-manage distress – does the internet have a role? A qualitative study

**DOI:** 10.1186/s12875-018-0874-7

**Published:** 2018-11-29

**Authors:** Alice Moult, Heather Burroughs, Tom Kingstone, Carolyn A. Chew-Graham

**Affiliations:** 1 Research Institute for Primary Care & Health Sciences, Keele University, Keele, Staffordshire ST5 5BG UK; 2 South Staffordshire and Shropshire NHS Foundation Trust, Stafford, ST16 3SR UK; 3 West Midlands Collaboration for Leadership in Applied Health Research and Care, West Midlands, UK

**Keywords:** Older adults, Internet, Self-management, Distress, General practitioners

## Abstract

**Background:**

Anxiety and depression are common in older adults, but often under-recognised by GPs. Rather than perceiving themselves as suffering from anxiety or depression, older adults are more likely to self-identify as experiencing low mood, stress or distress. Older people may also feel responsible for managing their own mood problems. The Internet has the potential to support the self-management of distress through accessing health information or social support.

**Methods:**

This study was approved by Keele University’s ethical review panel. Older adults who self-identified as experiencing distress were recruited from community groups in the West Midlands, England. To generate data, ‘think-aloud’ methods (including storyboards and an extract from an online forum) were embedded within semi-structured interviews. Thematic analysis, incorporating constant comparison methods, were used for data analysis.

**Results:**

Data saturation was achieved after 18 interviews. All participants reported having access to the Internet, but only a few described using the Internet to obtain general information or to conduct online purchases. Most participants described barriers to Internet use which included: a lack of interest, knowledge and confidence, a fear of technology and no trust in social media sites. Facilitators of Internet use included family encouragement and attending community groups which taught computer use. Female participants reported valuing the social contact provided by attending such groups. The Internet was seen as a source of health information once a GP had diagnosed a physical problem, but was not considered a source of information about distress or mood problems. Participants did not use the Internet to access social support and described a preference for face-to-face communication.

**Conclusions:**

GPs need to understand how an individual patient utilises the Internet. GPs should explore the self-management strategies already employed by older adults experiencing distress and understand that directing these older people to online support might not be acceptable. Encouraging distressed older adults to attend computer group classes might be useful as this permits face-to-face social contact, and may help to facilitate Internet use in the future.

**Electronic supplementary material:**

The online version of this article (10.1186/s12875-018-0874-7) contains supplementary material, which is available to authorized users.

## Background

Older adults have been typically defined as anyone who is 65 years old and above [1, 2], research has shown that anxiety and depression are common, but often unrecognised and untreated in individuals within this age group [3]. Older adults may not present mental health problems in primary care settings due to a myriad of patient-level barriers to care such as stigma, or an attribution of symptoms of mental health problems to the ageing process or physical comorbid conditions [4–8]. A lack of detection by General Practitioners (GPs) may also prevent older adults from receiving a diagnosis of a mental health problem [9]. If recognised by a GP, the management options offered may not be acceptable to older people [10].

Older adults are more likely to perceive themselves as experiencing low mood, stress or distress, rather than depression or anxiety [11]. Distress, depression and anxiety are related but distinct constructs [12]. Distress has been conceptualised as ‘the effort people must apply to maintain their psychosocial homeostasis and social functioning when experiencing stress’ [13]. Stress is often triggered by a range of life events such as bereavement or receiving a diagnosis of a physical health problem [14]. Distress can be comprised of symptoms including feeling low, worrying, irritability, poor concentration and sleep problems [13]. Depression includes all symptoms indicating distress, however may be identified by additional symptoms such as anhedonia, thoughts of self-harm, suicidal ideation and pervasive negative thoughts about self. In terms of anxiety, distinguishing symptoms from distress include irrational fears and avoidance behaviours [15]. Therefore, distress overlaps with depression and anxiety as an individual reaching the diagnostic criteria for these mental health problems is likely to have experienced distress [12].

Patient and practitioner barriers to help-seeking may result in older adults managing their own mood problems [16]. Self-management is defined as ‘taking increased responsibility for one’s own health, behaviour and well-being’ [17]. Effective self-management encompasses the ability to monitor one’s own health problems and to have the cognitive, behavioural and emotional responses necessary to maintain a satisfactory quality of life [18]. Policy initiatives support GPs encouraging older adults to self-manage both physical and mental health problems [19, 20].

To self-manage symptoms of depression and anxiety, younger adults employ several self-management strategies [21]. Some of these self-management strategies include using the Internet as a source of health information, or as a means of accessing social support [22–25]. Younger adults accessed social support through the Internet by engaging in social media sites [26], or within online forums [27]. Online forums are discussion sites where people can anonymously hold conversations in the form of posted messages. The anonymity afforded by online forums may lead to people being more open about their emotions [28].

Older adults could use the Internet to self-manage distress, however there is a lack of research which explores this. The Internet could be a source of information about distress, or be used in a way that enables distressed older adults to access social support. We report a study which explores the role of the Internet, particularly online forums, in how older adults self-manage distress.

## Methods

### Study design

A qualitative approach was adopted to explore the role of the Internet in how older adults self-manage distress. Ethics approval was obtained from Keele University’s ethical review panel (ERP1279). Written consent was obtained from all participants.

### Sampling strategy and recruitment

Recruitment to this study, which aimed to explore the role of the Internet in how older adults self-manage distress, was conducted through existing community groups in the Midlands, England. Stigma, or an attribution of symptoms of mental health problems to the aging process or physical illnesses, may prevent older adults from seeking help from healthcare services for their mood problems [4–8]. Recruiting from community groups enabled access to older adults who may not have presented their distress within primary care settings.

Inclusion criteria were used to ensure the sample consisted of individuals who were 65 years or older and who self-identified as distressed. To recruit distressed older adults, information sheets and flyers were given out within community groups (by AM). Distress was described on the information leaflets and flyers as feeling ‘low’, ‘stressed’, ‘anxious’ or ‘depressed’. The information sheets and flyers also stated that participants had to currently self-identify as distressed, or have been through an experience of distress within the past twelve months*.* Participants were also asked to describe their experiences of distress within the interview. A purposive sampling strategy was operated to ensure the sample consisted of a mixture of participants from various demographic backgrounds (see Table 1). AM tried to encourage snowball sampling [30] to recruit older adults who did not attend community groups. Older adults who attended the community groups were given additional information leaflets and were asked to pass them onto friends who might be interested in participating in the study.

### Data generation

Interviews were conducted (by AM) between September 2016 and March 2017. Interview topic guides were developed with the relevant literature and members of a research advisory group made up of older adults with mood problems; this ensured the wording of the prompts were acceptable to study participants. Semi-structured interviews were used to collect the data as they were sufficiently structured to address dimensions of the research question, but also permitted participants to offer new meanings to the topic under study. Embedded within the semi-structured interviews were ‘think aloud’ methods [29]. To ensure the ‘think aloud’ methods were suitable for potential participants, they were also created with advice from a research user group. Participants were first asked to read and reflect upon a transcript taken from an online forum (see Additional file 1). The transcript was taken from an open-access online forum which aimed to provide support for people experiencing depression. This was the only open-access online forum found by AM in which an individual described being an older person experiencing a mood problem. This older person received advice from several other users within the online forum. By showing participants the transcript from an online forum, this aimed to elicit discussion on using the Internet as a self-management strategy of distress. Participants were also asked to read and reflect upon two separate ‘storyboards’ as ‘think aloud’ activities. One ‘storyboard’ depicted an older adult having a positive encounter within an online forum (see Additional file 2). The second ‘storyboard’ depicted an older person having a negative encounter with an online forum (see Additional file 3). The storyboards also aimed to stimulate discussion around the Internet as a self-management strategy of distress. Contrasting the transcript from an online forum, the storyboards give some context into how a distressed older adult may come to use online forums.

Interviews were digitally recorded, transcribed and anonymised. Data collection and analysis were simultaneous and during this process the topic guide was modified to explore emerging areas of interest. Thematic analysis, incorporating constant comparison techniques, were used to analyse the data [30]. This method of analysis captured developing and recurrent themes within and between transcripts. Data analysis was conducted by four researchers (AM, HB, TK, CCG) from various disciplinary backgrounds, this increased the trustworthiness of the analysis [31]. Transcripts were transcribed and analysed independently by AM. Emerging codes and themes were discussed as a team until a consensus was achieved. Recruitment ceased once data saturation was reached.

## Results

All participants were recruited through community groups, the study did not manage to recruit any older people through snowballing methods. All participants were given a choice of a preferred venue in which to be interviewed (within the community group building or at home). Each participant chose to be interviewed within a private room at the building where the community groups were held. Interviews lasted between 44 and 92 min (mean 63 min).

### Sample characteristics

Eighteen older adults were interviewed (11 females, 7 males) with a mean age of 77.5 years (range 65–91). All participants reported having Internet access within their own homes (Table 1).

**Table 1:** Participant demographic profiles

Participant	Gender	Aged Between	Retired	Marital Status
1	Female	85–91	Yes	Widowed
2	Female	65–74	Yes	Married
3	Female	75–84	Yes	Divorced
4	Female	85–91	Yes	Widowed
5	Female	75–84	Yes	Widowed
6	Female	65–74	No	Married
7	Female	75–74	Yes	Married
8	Female	85–91	Yes	Married
9	Female	75–84	Yes	Married
10	Female	65–74	Yes	Married
11	Female	65–74	Yes	Married
12	Male	65–74	Yes	Married
13	Male	95–91	Yes	Married
14	Male	75–84	Yes	Married
15	Male	65–74	Yes	Married
16	Male	65–74	Yes	Widowed
17	Male	75–84	No	Widowed
18	Male	85–91	Yes	Married

### Presentation of themes

Key themes presented in this paper include: familiarity with the Internet, increasing familiarity, the Internet as a medical encyclopaedia and meeting social needs on the Internet. Illustrative data is given and identified by a participant number. It is also noted if the data was generated during the ‘think aloud’ activities.

### Familiarity with the internet

All participants were familiar with the concept of the Internet. One participant described accessing the Internet in the following way:

*“Yeah, we have Internet on our phones, iPad and computer yeah, everything. I've kept myself up to date but sometimes I have to send for the grandchildren [laughs] Can't do this, sometimes I get mixed up on my phone, its so small!”* [Participant 10]

Although some participants had access to the Internet through a range of devices, only a small number of participants were familiar in using the resource. These participants used the Internet as a source of general information:

*“You need to be told erm erm oh things like […] what is the weather like where you are, you know, oh it is sun-shining or it is raining.”* [Participant 3, generated from Storyboard 2]

A few participants reported using the Internet to conduct online purchases, which supported a sense of autonomy:

*“I love Amazon because I can't get out and shop without someone taking me and I get tired and very confused quickly so I end up buying the wrong thing, but on Amazon I can sit quietly and do it.”* [Participant 11]

Participants who engaged with the Internet used the resource for practical purposes.

Most participants were unfamiliar in using the Internet, particularly as a source of social interaction (e.g. using social media platforms or online forums):

*“Never been on one, never been on a chat forum so I can't comment really.”* [Participant 10]

Some participants reported a lack of knowledge about what the Internet might offer them:

*“I still have my computer in the corner since last Christmas and I've hardly used it since, I think you can take that as an answer (laughs) I'd never ever resort to the Internet because it’s, because it’s an unknown country, it is Greek to me.”* [Participant 13]

Due to a lack of familiarity in knowing how to use the Internet, participants preferred to self-manage by utilising alternative resources. The use of the word “*resort*” also alludes to a sense of fear, which is also echoed by Participant Fifteen:

*“The older you are because you're not au fait with this technology you are behind, you're in a different world and we're frightened of it.”* [Participant 15]

The source of fear seems rooted in many participants’ senses that the Internet is an “*unknown country*” or a “*different world*” – a territory they are not familiar with.

Many participants did not perceive older adults as a generation of Internet users and described how the Internet was unavailable to them when they were younger, as reported by Participant Six:

*“I think if we'd have been raised with them, I mean my son is a computer man because it is the jobs and the daughter-in-law is and grand-children, that is their jobs, and that is how you learn today, but we never did, and I think that is why I'm not interested.”* [Participant 6]

### Increasing familiarity

Although most participants were unfamiliar with using the Internet, some acknowledged factors which could encourage Internet use. One participant described that her family and computer group classes, specifically for older adults, contributed to her learning how to use the Internet:

*“Well after my Son-in-law got this computer, which was a big thing, the children tried to teach me but they aren't there and if things went wrong I had to wait for them to come again but then at my local school erm the council were putting some computer courses on during the day, mostly for older people, so I went to erm one and I learnt there that was a 6 week course and then there was one or two courses following on which were helpful.”* [Participant 4]

To learn how to use the Internet computer group classes were important, especially as some participants did not wish to solely depend on family members to teach them. Computer group classes increased a few participants’ confidence when engaging with the Internet:

*“Coming here has helped, we're more confident with it now, things don't scare me anymore now with it from coming here, yeah it doesn't scare me because coming here helps.”* [Participant 12]

An increase in confidence helped participants to overcome a fear of engaging with the Internet. One participant described that although she did not currently attend computer group classes, she would like to participate in the future:

*“I'd love to go to a computer class! Because I would like to learn about it but they are all in the evenings and I don't go out very much in the evenings and elderly people don't go out very much in the evenings.”* [Participant 3]

Attending computer group classes facilitated engagement with the Internet as such classes provided the support to learn and to increase participants’ familiarity with the Internet. Male participants valued attending computer classes for the purpose of learning a skill. Female participants also appreciated the social contact permitted by attending computer group classes:

*“More social, yeah, that is what that computer place is, I mean some of them are really into it and interested but not me […] I don't really want to know, I talk there.”* [Participant 6]

Computer classes served both an educational and social role for participants.

### The internet as a medical encyclopaedia

Some participants perceived that the Internet provided a wide-array of health information that was irrelevant to their needs:

*“It gives you too much information, far too much information and you don't need all of that information, you don't need to be told that you are this, you are that, you are the other.”* [Participant 3]

Access to a wide-range of information could have resulted in an older adult perceiving that they are suffering from an illness they did not have:

*“Won’t look up nothing on the Tinternet, will not look up illnesses on there because it can make you worse than when you bloody started, you're a bit paranoid really aren't you? You see all of these things and say I've got that.”* [Participant 5]

Participants suggested that a diagnosis should be made by a healthcare professional before the Internet would be used as a source of health information:

*“I like to know what is coming like with this operation, it is a bowel resection, and I want to know what is going to happen and why, so yes I do look. It is probably better to have a diagnosis before looking things up because you can give yourself everything so I wait to be told what it is so then I'll Google.”* [Participant 11]

Some participants reported using the Internet as a source of health information for physical illnesses and treatment options; this enabled participants to be prepared for what the treatments might entail. However, most participants suggested that the Internet was not an appropriate source of health information about distress, Participant Twelve reflected upon his wife’s physical illnesses when discussing this:

“*It is alright sending people to the Internet like to [my wife] they've said just check such and such thing on the Internet, like exercises for a bad back and things like that, more what you'd call physical I guess but not stuff like that.”* [Participant 12, generated from Storyboard 2]

Unlike physical health problems, some participants perceived distress as “*shameful*”:

*“Like having your leg off or you know tuberculosis or erm, you know, sort of diabetes, I mean people will brag about having things like that but not mental health, no, somehow it has become shameful.”* [Participant 1]

The concern regarding stigma drove many participants to assert that they were not experiencing a mental health problem:

*“I think most older adults would be in denial of stuff like that, I wouldn't see myself as having a problem like anxiety or stuff, they don't think that they'd have a problem so they'd just get on with life.”* [Participant 15]

Most participants wanted to manage their mood problems on their own. By not seeking help from healthcare services, participants are less likely to receive a diagnosis of a mental health problem. Even if participants sought help from healthcare services, distress may not warrant a clinical diagnosis and a diagnosis was needed before the Internet was used as a source of health information.

### Meeting social needs on the internet

A majority of participants described their concerns with social media sites, associating them with argumentative behaviours:

*“There is a lot of rows and […] a lot of unpleasantness going on Facebook and Twitter and stuff so that is my only objection to it, so no I won't use it.”* [Participant 11]

One participant noted that people may use social media to upload material which might be upsetting to them:

*“I know people who have said that they've done Facebook and then people have put something on which would upset me quite a bit, normally to do with animals and I thought no I don't want to see that.”* [Participant 2, generated from transcript taken from online forum]

Seeing upsetting material, or argumentative behaviours, on social media platforms may add to some participants’ feelings of distress. Many participants had no personal experience of using social media platforms. Negative perceptions of social media sites stemmed from hearing their friends’ experiences with using such platforms:

*“[My friend] told me this, she said she did [Facebook] to keep in touch with her daughter, but she said that there was a lot of negative things on, so I won't be involved for that reason nothing else.”* [Participant 11]

Hearing negative stories about social media contributed to why participants associated social media with negative online content.

Online forums in particular were not seen as a means of accessing social support. Many participants reported that it would be unlikely that older adults would engage with such platforms:

*“I think whatever you find on the computer, there is certain people that would use it, but […] erm I wouldn't and a lot wouldn't, nine out of ten wouldn't, I wouldn't even think of it.”* [Participant 18, generated from transcript taken from online forum]

Some participants described that they would not engage with online forums as they did not know the other online forum members, as described by Participant Five:

*“I know there is a person on the other side but you know, but you don't know who it is. People are far better, to go and talk to a person that is actually sitting there.”* [Participant 5, generated from Storyboard 1]

Not knowing who the other online forum members were prevented some participants from trusting the other members:

*“It isn't trustworthy you get cranks in everything don't you [laughs] that is why I don't go on because talking to a complete stranger.”* [Participant 10, generated from transcript taken from online forum]

This lack of trust suggested that online forums did not meet participants’ social needs. Instead, participants preferred face-to-face communication when discussing their distress, as described by Participant Five:

*“I think that one, Person Two, that is typical of people my age, we do get mood swings and we've just got to fight through it! You're better off speaking to a friend rather than this, rather than talking to a machine as I always say [laughs]”* [Participant 5, generated from transcript taken from online forum]

A preference for face-to-face communication permitted in-person social contact with friends. Participants viewed in-person social contact as important as it resulted in building and maintaining social networks and permitted access to social support.

## Discussion

To the best of our knowledge, this is the first study which explores older people’s views on using the Internet to manage distress. Each participant had access to the Internet within their home, but few engaged with the resource. A small number of participants reported using the Internet to obtain general information, or to conduct online purchases. Many participants alluded to a lack of knowledge about the Internet, or a fear of engaging with the resource, and did not perceive themselves as a generation of Internet users.

To overcome the unfamiliarity of using the Internet, participants reported the benefits of attending computer group classes which are specifically designed for older adults. Goll, Charlesworth, Scior and Stott [32] found that negative life events (e.g. physical health problems and bereavements) have been shown to reduce an older person’s interest in attending group activities. However, attending computer group classes have been linked to a reduced number of depressive symptoms reported by older people as they felt empowered by learning how to use the Internet [33]. This study supports this finding as male participants particularly valued attending computer group classes to learn a skill. Female participants also appreciated the face-to-face social contact afforded by attending such groups.

Younger adults have been reported to seek mental health information online [24]. In contrast, this study suggests that older adults were reluctant to seek information through the Internet for their mood problems. In line with Aref-Adib et al’s [24] findings, this study confirms that older people do seek physical health information online. Yet, the present findings stress the importance of receiving a diagnosis before physical health information was sought online; past literature did not identify the importance of this [24]. Participants did not perceive the Internet as a source of information about their mood problems. Unlike physical illnesses, participants perceived distress as “*private*” which prevented them from seeking help from healthcare services and possibly receiving a diagnosis of a mental health problem. To add to that, symptoms of distress may not warrant a diagnosis of a mental health problem and a diagnosis was needed before the Internet was seen as a source of health information.

When self-managing distress, participants described a preference for face-to-face communication as this permitted in-person social contact with friends. Younger adults use social media platforms and online forums as a means of accessing social support [25, 26]. This study found that older adults associate social media sites with arguments and upsetting material, this prevents older people from engaging with such platforms as a means of accessing social support. Moreover, seeing upsetting material and argumentative communication could add to an older person’s feelings of distress. Seraj [27] suggested that the anonymity afforded by online forums encouraged individuals to discuss their emotions. Participants in this study did not perceive such anonymity positively and suggested that online forums were untrustworthy due to their anonymous nature.

### Strength and limitations of the study

The study’s sample was diverse in certain aspects and captured both male and female older adults who had a wide age range. The study recruited from community groups which permitted access to participants who may not have presented their distress within primary care settings. However, all participants were recruited through community groups. Distressed older adults who do not attend community groups may have had different experiences of using the Internet. Another limitation of this study is that the sample did not capture older adults who did not have access to the Internet, which is a barrier to Internet use in itself. Although all participants had access to the Internet, the type of Internet access (e.g. high speed broadband) was also not explored.

## Conclusions

The findings infer that GPs should explore self-management strategies already being employed by distressed older adults as directing these older people to the Internet may not be acceptable. In order to overcome unfamiliarity, and to facilitate Internet use in the future, older adults should be encouraged to attend computer group classes. Through computer group classes, third sector services could explore the ways in which older adults could use the Internet to support mental health (e.g. by being a source of information or a means of accessing social support).

## Additional files

Additional file 1: Transcript from online forum. This file shows the transcript from an online forum which was used as a ‘think aloud’ activity. (DOCX 15 kb)

Additional file 2: Storyboard one. This file shows the first storyboard that was shown to participants and used as a ‘think aloud’ activity. (DOCX 467 kb)

Additional file 3: Storyboard Two. This file shows the second storyboard that was shown to participants and used as a ‘think aloud’ activity. (DOCX 545 kb)

## Abbreviations

GP: General Practitioner

## References

[1] Osmanovic-Thunström A, Mossello E, Åkerstedt T, Fratiglioni L, Wang HX (2015). Do levels of perceived stress increase with increasing age after age 65? A population-based study. Age Ageing.

[2] Chatterji S, Byles J, Cutler D, Seeman T, Verdes E (2015). Health, functioning, and disability in older adults—present status and future implications. Lancet.

[3] Bland P (2012). Tackling anxiety and depression in older people in primary care. The Practitioner.

[4] Kovandžić M, Chew-Graham C, Reeve J, Edwards S, Peters S, Edge D (2011). Access to primary mental health care for hard-to-reach groups: from ‘silent suffering ‘to ‘making it work’. Soc Sci Med.

[5] Burroughs H, Lovell K, Morley M, Baldwin R, Burns A, Chew-Graham C (2006). Justifiable depression’: how primary care professionals and patients view late-life depression?. A qualitative study Family Practice.

[6] Chew-Graham C, Kovandžić M, Gask L, Burroughs H, Clarke P, Sanderson H, Dowrick C (2012). Why may older people with depression not present to primary care? Messages from secondary analysis of qualitative data. Health & Social Care in the Community.

[7] Alderson SL, Russell AM, McLintock K, Potrata B, House A, Foy R (2014). Incentivised case finding for depression in patients with chronic heart disease and diabetes in primary care: an ethnographic study. BMJ Open.

[8] Coventry PA, Hays R, Dickens C, Bundy C, Garrett C, Cherrington A, Chew-Graham C (2011). Talking about depression: a qualitative study of barriers to managing depression in people with long term conditions in primary care. Fam Pract.

[9] Cepoiu M, McCusker J, Cole MG, Sewitch M, Belzile E, Ciampi A (2008). Recognition of depression by non-psychiatric physicians—a systematic literature review and meta-analysis. J Gen Intern Med.

[10] Zivin K, Kales HC (2008). Adherence to depression treatment in older adults. Drugs Aging.

[11] Wetherell JL, Petkus AJ, McChesney K, Stein MB, Judd PH, Rockwell E, Sewell DD, Patterson TL (2009). Older adults are less accurate than younger adults at identifying symptoms of anxiety and depression. J Nerv Ment Dis.

[12] Geraghty AW, Stuart B, Terluin B, Kendrick T, Little P, Moore M (2015). Distinguishing between emotional distress and psychiatric disorder in primary care attenders: a cross sectional study of the four-dimensional symptom questionnaire (4DSQ). J Affect Disord.

[13] Terluin B, van Marwijk HW, Adèr HJ, de Vet HC, Penninx BW, Hermens ML, van Boeijen CA, van Balkom AJ, van der Klink JJ, Stalman WA (2006). The four-dimensional symptom questionnaire (4DSQ): a validation study of a multidimensional self-report questionnaire to assess distress, depression, anxiety and somatization. BMC Psychiatry.

[14] Cromby J, Harper D, Reavey P. Psychology, mental health and distress. London: Macmillan. International Higher Education. 2013:9–21.

[15] Terluin B, Oosterbaan DB, Brouwers EP, van Straten A, van de Ven PM, Langerak W, van Marwijk HW (2014). To what extent does the anxiety scale of the four-dimensional symptom questionnaire (4DSQ) detect specific types of anxiety disorder in primary care? A psychometric study. BMC psychiatry.

[16] Khan N, Bower P, Rogers A (2007). Guided self-help in primary care mental health. Br J Psychiatry.

[17] Department of Health (2006). Supporting people with long term conditions to self-care.

[18] NHS England (2013). Improving general practice – a call to action.

[19] NHS England (2014). Five year forward view.

[20] Miller WR, Lasiter S, Ellis RB, Buelow JM (2015). Chronic disease self-management: a hybrid concept analysis. Nurs Outlook.

[21] Ramirez JL, Badger TA. Men navigating inward and outward through depression. Arch Psychiatr Nurs 2014;28:21–28. 10.1016/j.apnu.2013.10.001 24506983

[22] Grieken RA, Kirkenier AC, Koeter MW, Nabitz UW, Schene AH (2015). Patients' perspective on self-management in the recovery from depression. Health Expect.

[23] Villaggi Benjamin, Provencher Hélène, Coulombe Simon, Meunier Sophie, Radziszewski Stephanie, Hudon Catherine, Roberge Pasquale, Provencher Martin D., Houle Janie (2015). Self-Management Strategies in Recovery From Mood and Anxiety Disorders. Global Qualitative Nursing Research.

[24] Aref-Adib G, O’Hanlon P, Fullarton K, Morant N, Sommerlad A, Johnson S, Osborn D (2016). A qualitative study of online mental health information seeking behaviour by those with psychosis. BMC Psychiatry.

[25] Gowen K, Deschaine M, Gruttadara D, Markey D (2012). Young adults with mental health conditions and social networking websites: seeking tools to build community. Psychiatric Rehabilitation Journal.

[26] Webb M, Burns J, Collin P (2008). Providing online support for young people with mental health difficulties: challenges and opportunities explored. Early Intervention in Psychiatry.

[27] Seraj M (2012). We create, we connect, we respect, therefore we are: intellectual, social, and cultural value in online communities. J Interact Mark.

[28] Given LM (2008). The sage encyclopedia of qualitative research methods.

[29] Ericsson KA, Simon HA (1998). How to study thinking in everyday life: contrasting think-aloud protocols with descriptions and explanations of thinking. Mind Cult Act.

[30] Lincoln YS, Guba EG (1985). Naturalistic inquiry.

[31] Henwood K, Pigeon N (1992). (1992) qualitative research and psychological theorizing. Br J Psychol.

[32] Goll JC, Charlesworth G, Scior K, Stott J (2015). Barriers to social participation among lonely older adults: the influence of social fears and identity. PLoS One.

[33] Shapira N, Barak A, Gal I (2007). Promoting older adults’ well-being through internet training and use. Ageing and Mental Health.

