# Supplementary material for: How older adults self-manage distress – does the internet have a role? A qualitative study
Source: BMC Fam Pract. 2018 Nov 29;19:185. doi: 10.1186/s12875-018-0874-7 (PMC6263534; doi:10.1186/s12875-018-0874-7)
Supplement: Supplementary file 1 — Transcript from online forum. This file shows the transcript from an online forum which was used as a ‘think aloud’ activity. (DOCX 15 kb) [file 12875_2018_874_MOESM1_ESM.docx]

**Transcript from an online forum**

A post from an open-access online forum called [www.cureyourdepression.com](http://www.cureyourdepression.com/), obtained January 2016.

- ***Person one:*** I've always been asocial and subject to mood swings. That's not to say that I don't every now and then swing back into feeling nice. But I never find anyone whose company I enjoy and I don't enjoy exercise or travel anymore. My days and nights are only filled with reading, researching banal matters on the internet, and watching T.V., all of which don't offer me much more than just the passage of time. I also don't trust or like seeing doctors, I have little faith in them.
- ***Person two:*** Living to your sixties and waking up every morning is something to be grateful for. Try reaching out and being kind.
- ***Person three*:** I have a sign posted on the wall that says, KEEP CALM AND CARRY ON.
- ***Person Four:*** My recommendation is to participate in Recovery International meetings. Back in 1937 Abraham Lowe was not seeing any gains in his patients using psychoanalytic therapy. He created a system of cognitive and behaviour therapy. His programme is still very effective. Look up Recovery International on the web. Find a meeting or an internet meeting if there are no meetings near you. Try about 10 sessions to know if it helps you. The group sessions are free. It is by far the most powerful system I know. Go to a meeting and read the literature. Good luck!
- ***Person Five:*** What helps me is reading the Bible. I believe true joy can be found regardless of age.
