# Supplementary figures and images for: How older adults self-manage distress – does the internet have a role? A qualitative study
Source: BMC Fam Pract. 2018 Nov 29;19:185. doi: 10.1186/s12875-018-0874-7 (PMC6263534; doi:10.1186/s12875-018-0874-7)

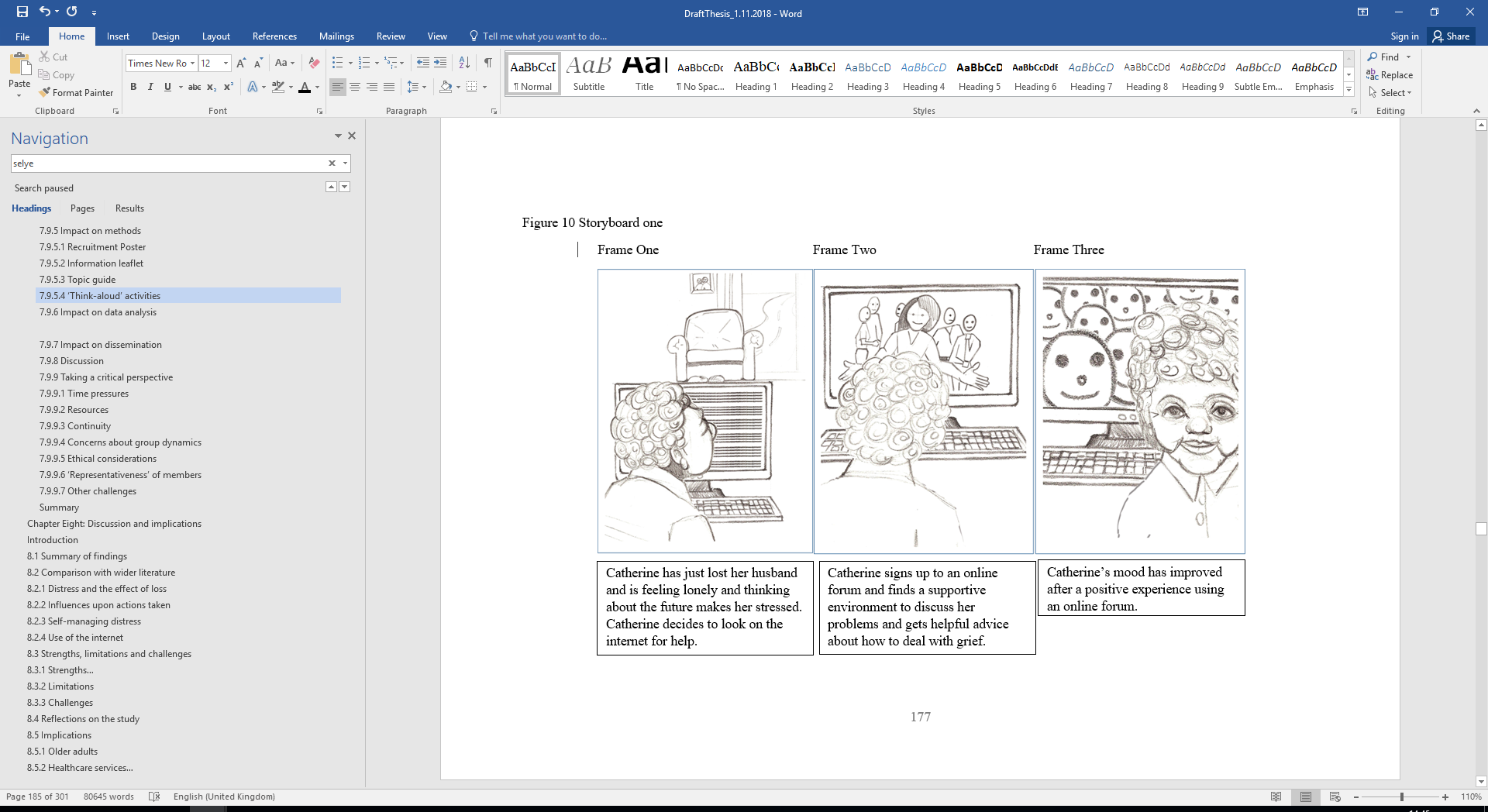

Supplement: Supplementary file 2 — Storyboard one. This file shows the first storyboard that was shown to participants and used as a ‘think aloud’ activity. (DOCX 467 kb) [file 12875_2018_874_MOESM2_ESM.docx]

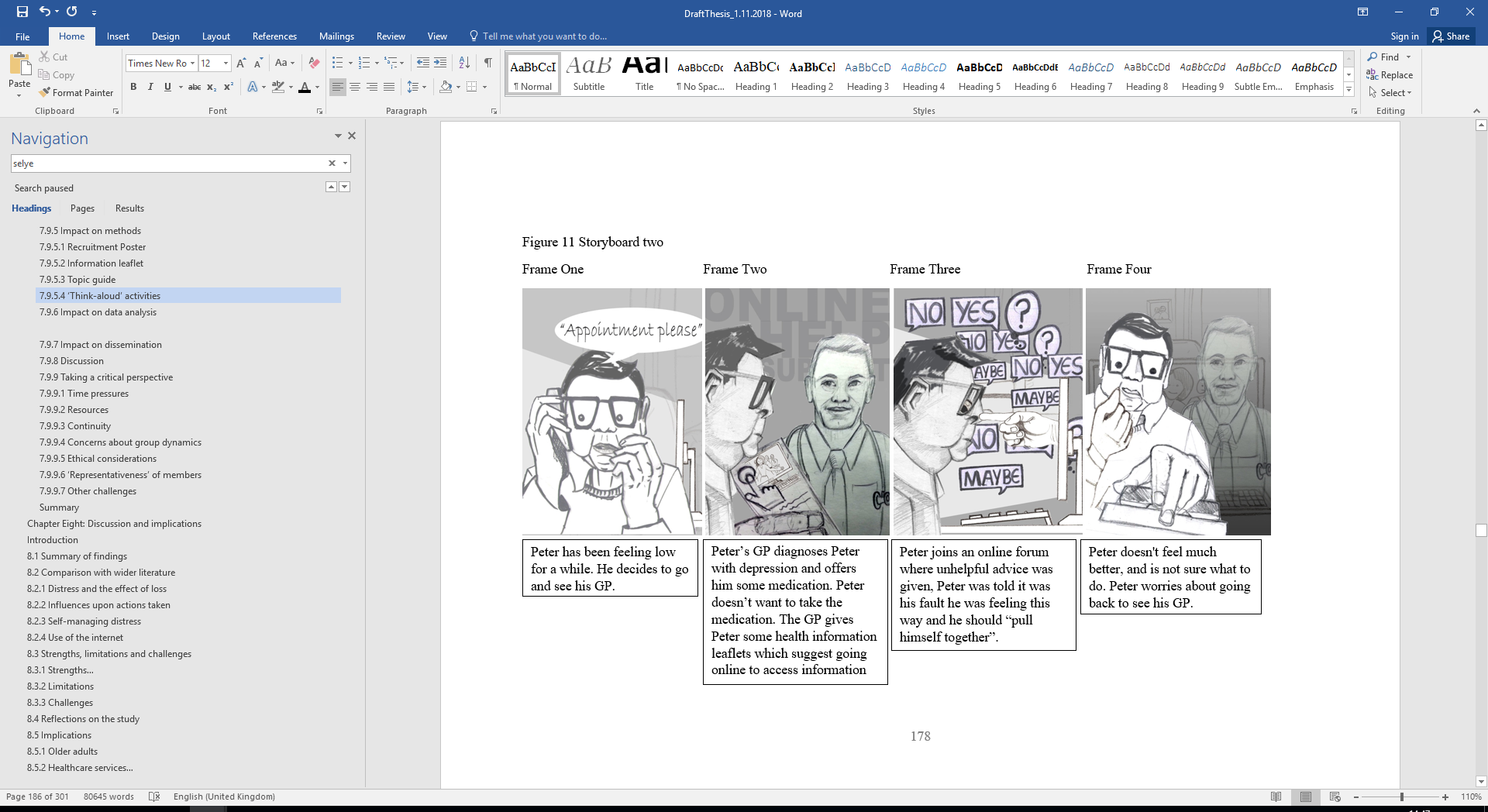

Supplement: Supplementary file 3 — Storyboard Two. This file shows the second storyboard that was shown to participants and used as a ‘think aloud’ activity. (DOCX 545 kb) [file 12875_2018_874_MOESM3_ESM.docx]
